# *Caenorhabditis elegans* ortholog of the p24/p22 subunit, DNC-3, is essential for the formation of the dynactin complex by bridging DNC-1/p150^Glued^ and DNC-2/dynamitin

**DOI:** 10.1111/j.1365-2443.2010.01451.x

**Published:** 2010-11

**Authors:** Masahiro Terasawa, Mika Toya, Fumio Motegi, Miyeko Mana, Kuniaki Nakamura, Asako Sugimoto

**Affiliations:** 1 Laboratory for Developmental Genomics, RIKEN Center for Developmental BiologyKobe, Hyogo 650-0047, Japan; 2 Center for Genomics and Systems Biology, Department of Biology, New York UniversityNew York, NY 10003, USA; 3 Department of Cell Biology, Research Institute for Microbiology, Osaka UniversitySuita, Osaka 565-0871, Japan; 4 Laboratory of Developmental Dynamics, Graduate School of Life Sciences, Tohoku University2-1-1 Katahira, Aoba-ku, Sendai 980-8577, Japan

## Abstract

Dynactin is a multisubunit protein complex required for the activity of cytoplasmic dynein. In *Caenorhabditis elegans*, although 10 of the 11 dynactin subunits were identified based on the sequence similarities to their orthologs, the p24/p22 subunit has not been detected in the genome. Here, we demonstrate that DNC-3 (W10G11.20) is the functional counterpart of the p24/p22 subunit in *C. elegans*. RNAi phenotypes and subcellular localization of DNC-3 in early *C. elegans* embryos were nearly identical to those of the known dynactin components. All other dynactin subunits were co-immunoprecipitated with DNC-3, indicating that DNC-3 is a core component of dynactin. Furthermore, the overall secondary structure of DNC-3 resembles to those of the mammalian and yeast p24/p22. We found that DNC-3 is required for the localization of the DNC-1/p150^Glued^ and DNC-2/dynamitin, the two components of the projection arm of dynactin, to the nuclear envelope of meiotic nuclei in the adult gonad. Moreover, DNC-3 physically interacted with DNC-1 and DNC-2 and significantly enhanced the binding ability between DNC-1 and DNC-2 *in vitro*. These results suggest that DNC-3 is essential for the formation of the projection arm subcomplex of dynactin.

## Introduction

The dynactin complex is an activator of cytoplasmic dynein, a major microtubule minus-directed motor protein. It is necessary to target dynein to specific cellular locations (Kardon & Vale 2009), to connect dynein to cargo (Holleran *et al.* 2001) and to increase processivity of the dynein motor activity (Karki & Holzbaur 1995; Vaughan & Vallee 1995; King & Schroer 2000). The dynein–dynactin complex is involved in a variety of cellular events, including membrane vesicle transport, mitotic spindle formation, centrosome separation and nuclear positioning (Kardon & Vale 2009).

Dynactin is a multiprotein complex of 1.2 MDa containing 11 different subunits and has two distinct structural domains; the projection arm that binds microtubules and motor proteins and the Arp1 rod that binds to cargo (Schroer 2004). The projection arm is composed of two copies of p150^Glued^ (DCTN1) and p24/p22 (DCTN3) and four copies of dynamitin (p50/DCTN2). The Arp1 rod contains an octameric polymer of the actin-related protein Arp1, the actin-capping protein CapZ α/β, another actin-related protein Arp11, p62 (DCTN4), p25 (DCTN5) and p27 (DCTN6) (Schroer 2004).

Among the three components of the projection arm, p150^Glued^ is the largest subunit and essential for the direct binding to microtubules and the dynein intermediate chain (Karki & Holzbaur 1995; Vaughan & Vallee 1995; Waterman-Storer *et al.* 1995). These binding activities are important for the effect of dynactin for the dynein processivity and microtubule anchoring at centrosomes (Quintyne *et al.* 1999; Quintyne & Schroer 2002). The second subunit, dynamitin, connects two structural domains of dynactin (Eckley *et al.* 1999). The smallest subunit of the dynactin complex, p24/p22, is the least conserved among the subunits (Karki *et al.* 1998; Pfister *et al.* 1998; Amaro *et al.* 2008). Human p24/p22 was shown to bind directly to p150^Glued^ and dynamitin *in vitro* (Karki *et al.* 1998; Maier *et al.* 2008). In budding yeast, the p24/p22 homolog was genetically shown to be essential for maintaining the association of p150^Glued^ with the dynactin complex (Amaro *et al.* 2008).

In the nematode *Caenorhabditis elegans*, some of the dynactin subunits have been identified based on the sequence similarity, and their functions were genetically analyzed. Loss-of-function phenotypes of DNC-1 (p150^Glued^), DNC-2 (Dynamitin), CAP-2 (CapZβ) and ARP-1 demonstrated that these proteins are required for microtubule-dependent events in early embryos, such as pronuclear migration, centrosome separation and mitotic spindle positioning (Skop & White 1998; Gonczy *et al.* 1999; Le Bot *et al.* 2003; Zhang *et al.* 2008). These phenotypes are similar to those by dynein knockouts (Gonczy *et al.* 1999). DNC-1, DNC-2 and ARP-1 localize to centrosomes during prometaphase, nuclear envelope, mitotic spindles, midbody remnants and P2-EMS cell borders (Skop & White 1998; Zhang *et al.* 2008; Ai *et al.* 2009). Other putative orthologs of dynactin components, except p24/p22, was detected in the *C. elegans* genome based on the amino acid sequence similarities, but their functions *in vivo* have not been well studied.

In this study, we genetically and biochemically demonstrate that W10G11.20/DNC-3 is the *C. elegans* ortholog of p24/p22, although this protein shows only a marginal sequence similarity to p24/p22 of other organisms. We also show that W10G11.20/DNC-3 is essential for the formation of dynactin complex by binding to both DNC-1 and DNC-2. Through this study, all the *C. elegans* dynactin subunits orthologs were biochemically confirmed to form a complex *in vivo*. Now that the complete subunits of dynactin were defined, *C. elegans* will provide an excellent model system to analyze the *in vivo* function and its regulatory mechanisms of the dynein–dynactin complex.

## Results

### W10G11.20 is required for microtubule-mediated events in early *C. elegans* embryos

In a large-scale protein–protein interaction analyses in *C. elegans* (Li *et al.* 2004), W10G11.20 was reported to interact with protein phosphatase 4 (PPH-4.1) that is required for centrosome maturation and establishment of chiasmata during meiotic prophase I (Sumiyoshi *et al.* 2002). The W10G11.20 gene encodes a protein with 171 amino acids containing coiled-coil structures, but a search by the Blast software did not detect any proteins with significant homologies.

To investigate the *in vivo* function of W10G11.20, the phenotypes by RNAi knockdown were analyzed. When maternal expression of W10G11.20 was inhibited by the soaking RNAi method (Maeda *et al.* 2001), the majority of embryos did not hatch (85%, 448/525), indicating that this gene is essential for embryonic development. To further analyze the function of W10G11.20, we investigated the phenotypes of live *W10G11.20*(*RNAi*) embryos using Nomarski microscopy.

**Figure 1:**
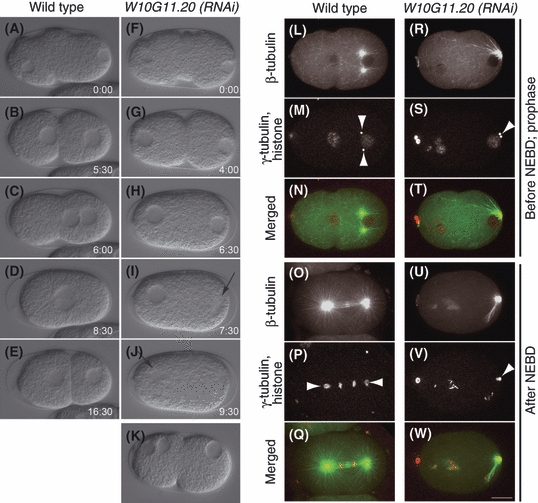
Early embryonic phenotypes in *W10G11.20*(*RNAi*) embryos. (A–K) DIC microscopy images of one-cell embryos. Time-series images of a wild-type embryo (A–E) and a *W10G11.20*(*RNAi*) embryo showing the defect of pronuclei meeting (F–J). (K) a *W10G11.20*(*RNAi*) embryo showing multiple female nuclei. Arrows in (I, J) indicate nuclear envelope break down (NEBD). (L–W) Confocal live images of embryos expressing GFP::β-tubulin, mCherry::γ-tubulin and mCherry::histone. (L–Q) Wild-type embryos. (R–W) *W10G11.20*(*RNAi*) embryos. (L–N, R–T) before NEBD. (O–Q, U–W) after NEBD. Arrowheads in (M, P, S, V) indicate centrosomes. Scale bar: 10 μm.

In the wild-type zygote, the male pronucleus locates to the posterior end, whereas the female pronucleus to the opposite side and migrates toward the male pronucleus (Fig. 1A,B). The pronuclei meet at approximately 70% of the embryo length (Fig. 1C), move to the center of the embryo and rotate 90° (Fig. 1D). Then, nuclear envelope breaks down and a bipolar spindle is assembled. In *W10G11.20*(*RNAi*) embryos, after the male and female pronuclei became visible (Fig. 1F), the migration of the male pronucleus did not occur (Fig. 1G,H). In some *W10G11.20*(*RNAi*) embryos, the female pronucleus partially migrated toward the male pronucleus, but never met with the male pronucleus. The nuclear envelope break down (NEBD) of the male pronucleus took place 1–2 min before that of the female pronucleus (Fig. 1I,J). After NEBD, bipolar spindle formation was not apparent by the DIC observation (Fig. 1J). In addition, *W10G11.20*(*RNAi*) embryos often had multiple female pronuclei (Table 1, Fig. 1K), indicating the defect of meiotic divisions. These phenotypes imply that W10G11.20 is required for microtubule-mediated events including pronuclear migration, bipolar spindle formation and oocyte meiosis. Therefore, we next examined the microtubule behaviors in *W10G11.20*(*RNAi*) embryos.

**Table 1:** Early embryonic RNAi phenotypes of *W10G11.20* and *dnc-2*

	Number of embryos analyzed	Embryos with single female pronucleus	Embryos with multiple female pronuclei	Average number of multiple female pronuclei†	Defect in male pronuclear migration‡	No apparent defect in one-cell stage
N2	13	13	–	–	–	13
*dnc-2*(*RNAi*)	21	16	5	2	20	1
*W10G11.20*(*RNAi*)	24	21	3	2	22	2

† Average number of multiple female pronuclei was calculated among the embryos with multiple female pronuclei.

‡ Male pronuclei fail to detach from cell cortex.

To concurrently visualize microtubules, centrosomes and chromosomes, we have constructed a strain that expresses GFP::β-tubulin, mCherry::γ-tubulin and mCherry::histone H2B (Toya *et al.* 2010). In the wild-type embryo, centrosomes separate to the opposite sides of the male pronucleus (Fig. 1M). In contrast, centrosomes in *W10G11.20*(*RNAi*) embryos did not separate and stay at the posterior to the male pronucleus (Fig. 1S). In the wild type, a bipolar mitotic spindle was observed after NEBD (Fig. 1P), but in *W10G11.20*(*RNAi*) embryos, it was never formed and two centrosomes stayed at the posterior side of the male pronucleus (Fig. 1V). These phenotypes are highly similar to those by the depletion of subunits of cytoplasmic dynein (DHC-1, dynein heavy chain) and dynactin (DNC-1/p150^Glued^, DNC-2/Dynamitin/p50, ARP-1/Arp1 and CAP-2/CapZβ) (Skop & White 1998; Gonczy *et al.* 1999; Le Bot *et al.* 2003) (Table 1). Based on these results, we speculated that W10G11.20 is likely to function with cytoplasmic dynein and/or dynactin.

### Localizations of W10G11.20 in early embryos are similar to those of dynein and dynactin subunits

To observe the subcellular localization of W10G11.20 in adult gonads and early embryos, we generated transgenic lines that express GFP::W10G11.20::FLAG under the control of the *pie-1* promoter that drives transcription in the germ-line. The FLAG-tag was included in the construct so that this transgenic line can also be used for affinity purification of W10G11.20-interacting proteins.

**Figure 2:**
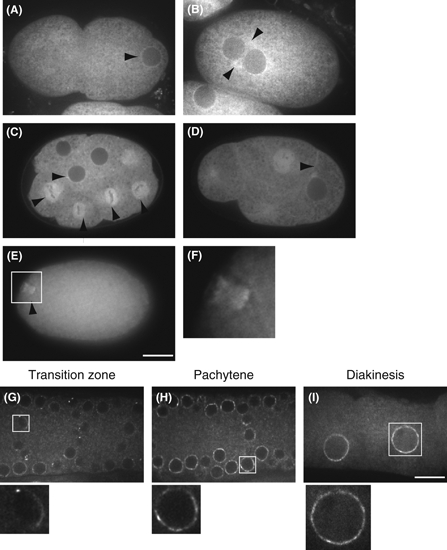
Subcellular localization of GFP::W10G11.20. (A–F) Early embryos. Arrowheads indicate the nuclear envelope (A), centrosomes (B, C), mitotic spindle (C), cell border (D) and meiotic spindle (E). Panel F shows magnified views of the indicated regions in panel E. (G–I) Adult gonads: transition zone (G), pachytene (H) and diakinesis (I) stages. Lower panels show magnified views of the indicated regions in upper panels. Scale bar: 10 μm.

Live imaging analysis of early embryos using a spinning-disk confocal time-lapse microscopy revealed that GFP::W10G11.20::FLAG was present in the cytoplasm throughout cell cycle (Fig. 2A–F). As the cell cycle proceeds, the signal at the nuclear periphery became brighter, and it was also detected around centrosomes (Fig. 2A–C). In metaphase, when the nuclear membrane becomes partially permeable, GFP::W10G11.20::FLAG was enriched on both sides of condensed chromosomes (metaphase plates) (Fig. 2C). After cell division, GFP::W10G11.20::FLAG was temporally enriched at the cell boundaries (Fig. 2D). In addition, GFP::W10G11.20::FLAG localized to the female meiotic spindle (Fig. 2E,F). These localization patterns are similar to those of the cytoplasmic dynein subunit DHC-1 and the dynactin subunits, DNC-1, DNC-2, ARP-1 (Skop & White 1998; Gonczy *et al.* 1999; Zhang *et al.* 2008).

### W10G11.20 localizes to the nuclear envelope of the adult germ cells

As some subunits of dynein and dynactin was shown to be present at the nuclear periphery in the *C. elegans* germ-line (Malone *et al.* 2003; Sato *et al.* 2009; Zhou *et al.* 2009), localization of W10G11.20 in the adult hermaphrodite gonads was observed using the transgenic line expressing GFP::W10G11.20::FLAG described earlier. While GFP::W10G11.20::FLAG was undetectable in the distal gonad where germ cells are mitotic, it was detected as bright dots at the periphery of nuclei (a few dots per nucleus) in the transition zone (Fig. 2G). After the pachytene stage, it localized to the entire periphery of the nuclei (Fig. 2H,I).

The localization of W10G11.20 at the nuclear periphery was also confirmed by antibody staining. We raised polyclonal antibodies against W10G11.20 and stained the gonads of wild-type adult hermaphrodites. The W10G11.20 staining coincided with that of KT23 monoclonal antibody that recognizes nuclear membrane (Takeda *et al.* 2008) (Fig. S1A,B in Supporting Information). This signal by anti-W10G11.20 antibody was not detected in the gonad of *W10G11.20*(*RNAi*) worms, confirming that this signal is specific for the W10G11.20 protein (Fig. S1C,D in Supporting Information). This localization at the nuclear periphery in adult gonads is similar to the reported localization of dynein heavy chain (DHC-1) and p150^Glued^ (DNC-1) (Malone *et al.* 2003; Sato *et al.* 2009; Zhou *et al.* 2009).

Taken together, the loss-of-function phenotypes and subcellular localization in the gonad and early embryos of W10G11.20 are highly similar to those of the subunits of the dynein–dynactin, strongly indicating that W10G11.20 functions closely with this complex.

### W10G11.20 is a component of the dynactin complex

Above-mentioned analyses raised the possibility that W10G11.20 may be a component of the dynein–dyn-actin complex, although no sequence similarity to any of the known subunits was detected. In *C. elegans*, 10 of 11 dynactin subunits were detected in the genome. Among them, DNC-1/p150^Glued^, DNC-2/Dynamitin, ARP-1/Arp1 and CAP-2/CapZβ were genetically characterized (Skop & White 1998; Gonczy *et al.* 1999; Le Bot *et al.* 2003). Six other subunits (Actin, CAP-1/CapZα, DNC-4/p62, Y71F9AL.14/p25, Y54E10A.5/p27 and C49H3.8/Arp11) were reported based on their sequence similarity to the corresponding subunits (Waddle *et al.* 1993; Eckley *et al.* 1999; Eckley & Schroer 2003) (Table 2). The only subunit whose ortholog unfound was p24/p22. Because the molecular mass of W10G11.20 (19.4k) is similar to p24/p22, we suspected that W10G11.20 might be the functional counterpart of p24/p22.

**Table 2:** Complete components of the *Caenorhabditis elegans* dynactin complex identified by mass spectrometry

# of fragment	*C. elegans* protein name†	Coverage (%)‡	Mammalian homologs	Blastp*E* value§	References
1	DNC-1	53	p150^Glued^ (DCTN1)	2e-104	Skop & White (1998); Le Bot *et al.* (2003)
4	DNC-2	50	Dynamitin (p50/DCTN2)	6e-15	Skop & White (1998)
2	W10G11.20 (DNC-3)	63	p24 (DCTN3)	Not significant	This study
3	DNC-4	26	p62 (DNTN4)	2e-43	Eckley *et al.* (1999)
3	C49H3.8 (ARP-11)	49	Arp11 (ACTR10)	6e-31	Eckley & Schroer (2003)
3,4,5	Actin	33	actin		
4	ARP-1	59	Arp1 (ACTR1A)	3e-162	Le Bot *et al.* (2003); Zhang *et al.* (2008)
5	CAP-1	50	CapZα	3e-81	Waddle *et al.* (1993)
5	CAP-2	35	CapZβ	1e-112	Waddle *et al.* (1993); Le Bot *et al.* (2003)
6	Y54E10A.5 (DNC-6)	46	p27 (DCTN6)	7e-29	Eckley *et al.* (1999)
6	Y71F9AL.14 (DNC-5)	25	p25 (DCTN5)	6e-30	Eckley *et al.* (1999)

† Genes named in this study are indicated with parentheses.

‡ Percent coverage in the mass spectrometric analysis of the immunoprecipitation shown in Fig. 3.

§ *E* values of Blastp against human orthologs.

**Figure 3:**
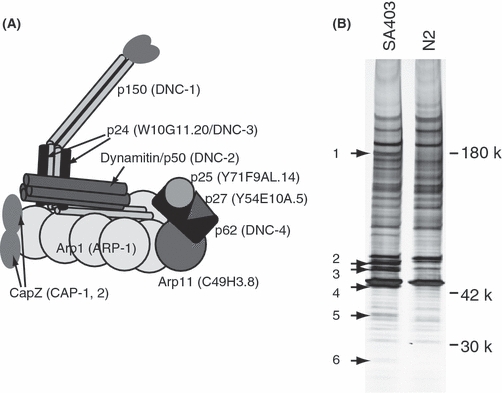
W10G11.20 is a component of the dynactin complex. (A) Diagram of the mammalian dynactin complex. *Caenorhabditis elegans* homologs are shown in parentheses. (B) Co-immunoprecipitation of the dynacitn subunits with FLAG-tagged W10G11.20. Silver stained 5%–20% gradient polyacrylamide gel is shown. Arrows indicate the proteins specifically precipitated from the extract of the GFP::W10G11.20::FLAG transgenic line, which were analyzed by mass spectrometry.

To test this possibility, we examined whether W10G11.20 directly interacts with other dynactin components. The extract from the transgenic worm that expresses GFP::W10G11.20::FLAG was immunoprecipitated using anti-FLAG antibody, and proteins co-precipitated with GFP::W10G11.20::FLAG were determined by mass spectrometry (Fig. 3). GFP::W10G11.20::FLAG and ten dynactin components described earlier(DNC-1, DNC-2, DNC-4, Y71F9AL.14/p25, Y54E10A.5/p27, ARP-1, C49H3.8/Arp11, CAP-1, CAP-2 and actin) were detected with a significant sequence coverage (Table 2). Furthermore, in reciprocal immunoprecipitation experiments using worm extracts (wild type, GFP::DNC-2 or GFP::ARP-1 transgenic lines) and antibodies against DNC-1 or GFP, co-precipitation of W10G11.20 with DNC-1, GFP::DNC-2 and GFP::ARP-1 was confirmed (Fig. S2 in Supporting Information). These results strongly suggest that W10G11.20 is a component of dynactin complex and that it is the ortholog of the p24/p22 subunit.

**Figure 4:**
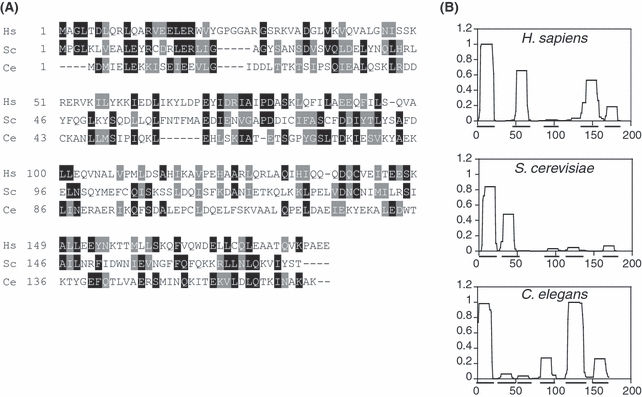
Sequence and structural features of W10G11.20 are similar to those of human p24/p22 and yeast Ldb18. (A) Multiple alignment of human p24/p22 (Hs), Ldb18 (Sc) and W10G11.20 (Ce). The alignment was generated by clustalW program. Identical residues are indicated by black boxes, and similar residues are shaded in gray. (B) p24/p22, Ldb18 and W10G11.20 have 5–6 coiled-coil domains. Coiled-coil structures were predicted using the COILS algorithm with a 14-residue window.

To further confirm that W10G11.20 corresponds to p24/p22, we compared the peptide sequences of W10G11.20 with human p24/p22 and the budding yeast p24/p22 ortholog, Ldb18 (Fig. 4). Low similarities that were missed by the Blast search were detected (18% and 16% identity, 39% and 43% similarity with W10G11.20, respectively), and all three proteins contain 5–6 coiled-coil domains (predicted using the COILS algorithm) in the similar locations (Fig. 4). Taken together, we concluded that W10G11.20 is the *C. elegans* ortholog of the dynactin component p24/p22 and named it DNC-3. [In addition, p25 ortholog Y71F9AL.14, p27 ortholog Y54E10A.5 and Arp11 ortholog C49H3.8 were named DNC-5, DNC-6 and ARP-11, respectively (Table 2)].

### DNC-3 is required for the localization of other dynactin components at the nuclear envelope

**Figure 5:**
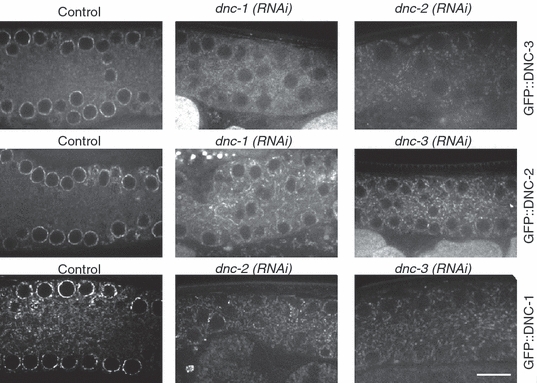
DNC-3/W10G11.20 is required for localization of DNC-1 and DNC-2 at the nuclear envelope in gonads. Confocal images of the pachytene stage region in adult gonads are shown. From top to bottom: images of a gonad expressing GFP::DNC-3, GFP::DNC-2 and GFP::DNC-1. The left panels show the gonads without RNAi treatment. The middle and right panels show images of gonads with *dnc-1* (*RNAi*), *dnc-2* (*RNAi*) or *dnc-3* (*RNAi*)*,* as indicated. Scale bar: 10 μm.

It was previously reported that dynein localizes to the periphery of germ cell nuclei and required for the arrangement of nuclei in the gonad (Zhou *et al.* 2009). We observed whether dynactin is also necessary for the gonad architecture. In the wild-type adult hermaphrodites, germ-line nuclei are arranged at the periphery of the gonad. In contrast, germ-line nuclei were disarrayed from the surface of the gonad in the *dnc-1*(*RNAi*), *dnc-2*(*RNAi*) and *dnc-3*(*RNAi*) worms (Fig. 5). These results demonstrated that the dynactin complex is required for the formation of regular arrays of germ-line nuclei to the surface of the gonads, which is consistent with the requirement of dynein in this process.

We next tested whether DNC-3 is necessary for the localization of other subunits of dynactin complex at the nuclear envelope. In untreated lines, GFP::DNC-1 and GFP::DNC-2 localized to the nuclear envelope of pachytene or diakinesis stage nuclei in the adult gonads. In contrast, their localization was largely impaired in *dnc-3*(*RNAi*) worms (Fig. 5). In the reciprocal experiments, GFP::DNC-3::FLAG did not localize to the nuclear envelope in *dnc-1*(*RNAi*) and *dnc-2*(*RNAi*) gonads (Fig. 5) and embryos (data not shown). These results demonstrated that localization of dynactin components DNC-1, DNC-2 and DNC-3 at the nuclear envelope are mutually dependent, implying that the targeting of the dynactin complex to the specific subcellular loci is determined not by a single subunit, but by a complete functional complex.

### DNC-3 physically interacts with DNC-2 and DNC-1 and connects them together

**Figure 6:**
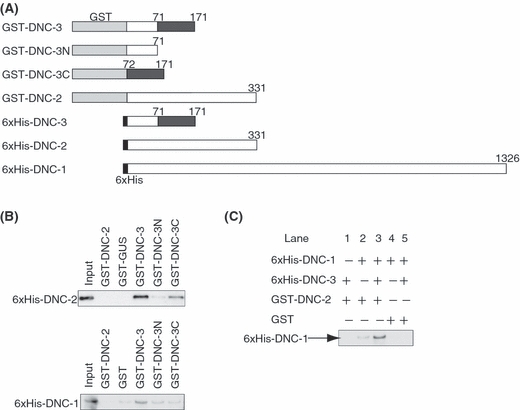
DNC-3/W10G11.20 physically interacts with DNC-1 and DNC-2. (A) Diagram of the constructed DNC recombinant proteins. Light gray and black bars indicate the GST and 6xHis tag, respectively. Dark gray bars indicate the C-terminal region of DNC-3. Numbers indicate amino acid. (B) Upper panel: Pull-down assay for 6xHis-tagged DNC-2 by GST-tagged proteins as indicated. Lower panel: Pull-down assay for 6xHis-tagged DNC-1 by GST-tagged proteins as indicated. (C) Pull-down assay for 6xHis-DNC-1 by either GST-DNC-2 or GST with or without 6xHis-DNC-3.

Mutually dependent subcellular localization among DNC-1, DNC-2 and DNC-3 and the co-immunoprecipitation results indicate that these proteins are likely to physically interact with each other. Direct interaction between DNC-1, DNC-2 and DNC-3 was examined *in vitro* using the recombinant proteins expressed in *Escherichia coli*. GST pull-down assays demonstrated that GST-DNC-3 directly interacts with 6xHis-DNC-1 and 6xHis-DNC-2 (Fig. 6B). Using the truncated versions of DNC-3 tagged with GST, we found that the C-terminal half of the DNC-3 was sufficient to interact with 6xHis-DNC-2, whereas both N-terminal and C-terminal regions of DNC-3 were required to interact with 6xHis-DNC-1 (Fig. 6B).

Our *in vitro* assay 6xHis-DNC-1 did not bind to GST-DNC-2 efficiently (Fig. 6B). Because DNC-3 bound to both DNC-1 and DNC-2, we speculated that DNC-3 connects both proteins to form a tertiary complex. To test this model, we examined the binding of DNC-1 and DNC-2 in the presence and absence of DNC-3. Whereas direct interaction of 6xHis-DNC-1 and GST-DNC-2 was very weak, the presence of 6xHis-DNC-3 dramatically enhanced the interaction (Fig. 6C lane 3). Taken together, we concluded that DNC-3 connects DNC-1 with DNC-2 efficiently to form the dynactin complex.

## Discussion

In *C. elegans*, while 10 of 11 dynactin subunits are detectable in the genome based on the sequence similarities (Eckley *et al.* 1999; Eckley & Schroer 2003), the ortholog of p24/p22 has not been clearly defined. Previously, W02A2.2 was reported as the candidate *C. elegans* ortholog of p24/p22 based on a Blast search result (Eckley & Schroer 2003), but its sequence similarity to p24/p22 was very low and no further supporting evidence have been presented. In this study, based on four lines of evidence, we concluded that DNC-3/W10G11.20 is the *C. elegans* ortholog of p24/p22. First, the knockout phenotypes of *dnc-3* were virtually equivalent to those of the known dynactin components. Second, the subcellular localization of DNC-3 coincided with other dynactin subunits. Third, DNC-3 co-immunoprecipitated with all other ten dynactin subunits and directly bound to DNC-1/p150^Glued^ and DNC-2/dynamitin. Finally, the overall size and secondary structure of DNC-3 is similar to those of human and yeast p24/p22. We analyzed all the major coprecipitated bands with DNC-3 by mass spectrometry, and no other uncharacterized proteins were identified. Collectively, although the similarity of primary amino acid sequence is marginal, DNC-3 is most likely to be the p24/p22 ortholog in *C. elegans*.

Our *in vitro* assay demonstrated that DNC-3 function as an adaptor to connect DNC-1 with DNC-2. Consistently, in budding yeast, disruption of the p24/p22 homolog *LDB18* results in reduction of Jnm1 (homolog of DNC-2/dynamitin)-Nip100 (homolog of DNC-1/p150^Glued^) interaction by approximately 95% (Amaro *et al.* 2008). It was also reported that mammalian p150^Glued^ (DNC-1 ortholog), p50 (DNC-2 ortholog) and p24/p22 (DNC-3 ortholog) form a stable complex, whereas direct interaction between p150^Glued^ and dynamitin was very weak or undetectable (Karki *et al.* 1998; Moore *et al.* 2008). Thus, the essential role p24/p22/DNC-3 in the assembly of a dynactin to link p150^Glued^/DNC-1 and dynamitin/DNC-2 is evolutionarily conserved.

In interphase in early embryos and meiotic prophase I in the adult gonads, dynactin localizes to the nuclear envelope. While bovine p150^Glued^ has the ability to bind to the nuclear pore complex by itself (Payne *et al.* 2003), our data showed that DNC-1, DNC-2 and DNC-3 are mutually dependent for their localization to the nuclear envelope. Thus, in *C. elegans*, none of these subunits are sufficient for localizing to the nuclear envelope by itself, and other components might be responsible. Notably, dynein/dynactin does not accumulate on the nuclear envelope in budding yeast and fission yeast, both of which apparently lack p62, p25 p27 subunits that form “the pointed-end complex” subdomain of dynactin (McMillan & Tatchell 1994; Kahana *et al.* 1998; Eckley & Schroer 2003). Therefore, one possibility might be that the pointed-end complex or any of its components may be involved in the nuclear envelope targeting in *C. elegans*.

In metazoans, the dynein–dynactin complex is involved in a variety of cellular events including centrosome separation, mitotic spindle formation and nuclear migration/positioning (Gonczy *et al.* 1999; Morris 2003). In addition, dynein–dynactin is required for efficient homologous chromosome paring during meiotic prophase in some organisms including fission yeast (Yamashita *et al.* 1997; Niccoli *et al.* 2004) and *C. elegans* (Sato *et al.* 2009). In the *C. elegans* germ cells undergoing homologous chromosome paring, dynein and nuclear envelope proteins ZYG-12 and SUN-1 form patch-like structures (Sato *et al.* 2009), which is similar to the ones observed for GFP::DNC-3::FLAG. Additionally, PPH-4.1 (protein phosphatase 4), a DNC-3 interactor identified by yeast 2-hybrid analysis (Li *et al.* 2004), is also reported to be required for meiotic recombination (Sumiyoshi *et al.* 2002). Currently, it is not well understood whether dynactin activity is regulated by protein phosphorylation, but it is an intriguing possibility that the phosphorylation state of DNC-3 is regulated by PPH-4.1. It will be of future interest to examine the interaction between these proteins in the process of homolog pairing and meiotic recombination.

While dynein/dynactin activity is essential for mitosis in metazoans including *C. elegans* and vertebrates, it is dispensable for mitosis in budding and fission yeast (McMillan & Tatchell 1994; Yamashita *et al.* 1997). In budding yeast, dynein/dynactin is required for nuclear positioning and short-range nuclear movement, but not for chromosome segregation (McMillan & Tatchell 1994). In fission yeast, the expression of dynein/dynactin is limited in meiosis (Yamashita *et al.* 1997). In addition to the difference in the requirement for mitosis, yeasts lack p62, p25 and p27 subunits of the dynactin complex. Although yeasts have been extensively used for genetic studies of dynactin, these differences in requirements and components imply that dynactin might have acquired new physiological roles and regulatory mechanisms in the course of evolution of metazoans. In this study, we revealed that all the dynactin components are conserved between *C. elegans* and mammals. Now that all the *C. elegans* dynactin subunits were defined, it will be more feasible to dissect the role of each dynactin component by combining biochemistry and genetics, for example, in the complex assembly and in the modulation of its activity. We believe that *C. elegans* will provide an excellent metazoan model system to study the *in vivo* functions and regulatory mechanisms of the dynein–dynactin complex.

## Experimental procedures

### Worm strains

The following strains were used: N2, wild type; SA403, *unc-119*(*ed3*)*;tjIs191*[*pie-1 promoter::gfp-TEV::dnc-3::FLAG unc-119*(*+*)]; SA155, *unc-119*(*ed3*)*;tjIs11*[*pie-1 promoter::gfp::dnc-2 unc-119*(*+*)]; WH257, *unc-119*(*ed3*)*;ojIs66*[*pie-1 promoter::gfp::dnc-1 unc-119*(*+*)]; WH258, *unc-119*(*ed3*)*;ojIs57*[*pie-1 promoter::gfp::dnc-2 unc-119*(*+*)]; WH259, *unc-119*(*ed3*)*;ojIs47*[*pie-1 promoter::gfp::arp-1 unc-119*(*+*)]; SA250, *unc-119*(*ed3*)*;tjIs54*[*pie-1 promoter::gfp::tbb-2 pie-1 promoter::2xmCherry::tbg-1 unc-119*(*+*)*;tjIs57*[*pie-1 promoter::mCherry::H2B unc-119*(*+*)] (Toya *et al.* 2010)*.*

### RNAi

RNAi was carried out by the soaking method (Maeda *et al.* 2001). dsRNA was prepared from cDNA clones, as described (Sumiyoshi *et al.* 2002). The cDNA clones (gifts from Yuji Kohara) used were as follows: yk1299g08 (*dnc-1*), yk705c10 (*dnc-2*). The following primers were used to amplify *W10G11.20/dnc-3* cDNA (T7 promoter sequences in bold). Forward, **TAATACGACTCACTATAGGG**ATGGACATGATCGAGCTTGA, reverse, **TAATACGACTCACTATAGGG**TTTAGCTTTTGCGTTGATTTTC. Worms were soaked in 2 mg/mL dsRNA solutions and incubated at 20 or 24 °C for 24 h. The worms were then cultured at 20 or 24 °C and observed 24 h after recovery from the dsRNA soaking.

### Construction of fluorescent strains

Construction of the strain SA250 expressing GFP::β-tubulin, mCherry::γ-tubulin and mCherry::Histone are described in (Toya *et al.* 2010). SA403 that expresses GFP-TEV::DNC-3::FLAG was constructed by high-pressure particle bombardment (Praitis *et al.* 2001) of the DP38 worms with the plasmid pMTN1G_W10G11.20*.* The plasmid was constructed as follows. The full length of W10G11.20 cDNA was cloned into pENTR/TEV/D-TOPO entry vector (Invitrogen, Carlsbad, CA, USA) using the following primers: forward, **CACC**ATGGACATGATCGAGCTTGA and reverse, TTA**TTTATCGTCATCGTCTTTGTAGTC**TTTAGCTTTTGCGTTGATTTTC. The forward primer has four additional bases right before the start codon for TOPO cloning (in bold). The reverse primer contains 24 bases coding FLAG-tag right before the STOP codon (in bold). The insert was recombined into the pMTN1G plasmid (Toya *et al.* 2010) by LR reaction.

### Live imaging microscopy

For observations of pronuclear movement and bipolar spindle formation, adult hermaphrodites in Egg buffer were dissected on coverslips, which were inverted onto 2% agarose pads and the embryos were analyzed by time-lapse DIC microscopy. Images were taken on Axioplan 2 (Zeiss, Carl Zeiss, Jena, Germany) microscope equipped with a CoolSNAP HQ CCD camera (Photometrics, Tucson, AZ, USA) using a 63×, 1.2 NA C-Apochromat objective lens.

SA250 strain was observed as previously described (Toya *et al.* 2010). For the observation of GFP-tagged DNC proteins in embryos, they were mounted on an agarose pads as described earlier. For observation of GFP-tagged DNC proteins in adult gonads, worms were paralyzed with 1 mg/mL of tetramisole in M9 buffer and mounted on an agarose pad. Embryos or gonads were observed with at 24 °C with an UPlanApo 100× 1.40 NA oil immersion lens by using a CSU-X1 spinning-disk confocal system (Yokogawa Electric Corp., Tokyo, Japan) mounted on an IX71 inverted microscope (Olympus, Tokyo, Japan). The specimens were illuminated with a 488 or 561 nm diode-pumped solid-state laser (25–35 mW; CVI Melles Griot). Images were acquired with an Orca-R2 12-bit/16-bit cooled CCD camera (Hamamatsu Photonics, Shizuoka, Japan), and the acquisition system was controlled by MetaMorph software (Molecular Devices, Inc., Downingtown, PA, USA). Fluorescence images were acquired by 2 × 2 binning in the camera and were processed with MetaMorph software and Adobe Photoshop (Adobe Systems Inc., San Jose, CA, USA).

### Immunoprecipitation and mass spectrometry

Young adults worms expressing GFP-TEV::DNC-3::FLAG were grown synchronously on EPP plates and bleached to obtain embryos. Embryos were washed in lysis buffer (50 mm Hepes at pH7.4, 1 mm EGTA, 1 mm MgCl_2_, 100 mm KCl, 10% glycerol and 0.05% NP40) and frozen in liquid N_2_. Approximately 3 millions of embryos were suspended in 1 mL of lysis buffer containing a protease inhibitor cocktail (Roche, Basel, Switzerland) supplemented with 1 mm PMSF and lysed by sonication. After centrifugation at 20 000 *g* for 10 min, 20 μg of anti-FLAG antibody preincubated with 50 μL of Protein G-dynabeads was mixed with the supernatant for 3 h at 4 °C. The immunoprecipitates were collected by magnets and washed with 1 ml of lysis buffer 3 times and eluted by 200 μm of FLAG peptides (Sigma Chemical Co., St Louis, MO, USA) for 90 min. All of the eluates were precipitated with acetone and lysed in SDS loading buffer, then electrophoresed on 5%–20% SDS–PAGE and visualized with silver staining. Bands specifically detected in the extract from the FLAG-tagged strain were cut out of the gel and analyzed by mass spectrometry.

### Protein structure analysis

Sequences of W10G11.20/DNC-3, yeast homolog of p24/p22, Ldb18 and human p24/p22 were aligned by clustalW (Thompson *et al.* 1994) (http://clustalw.ddbj.nig.ac.jp). The coiled-coil structures of those proteins were predicted by COILS algorithm (Lupas *et al.* 1991) (http://www.ch.embnet.org/software/COILS_form.html).

### Preparation of anti-W10G11.20/DNC-3 antibody

Anti-W10G11.20/DNC-3 rabbit serum was made against GST-fused N-terminal truncated W10G11.20/DNC-3 (49–171). The W10G11.20/DNC-3 fragment was amplified from a cDNA library and cloned into the entry vector pENTR201 (Invitrogen). The entry clone was recombined with pDEST15 (Invitrogen) by LR recombination. The obtained plasmid was used for protein expression in *E. coli*, and GST-W10G11.20/DNC-3 was purified using GST-sepharose (GE Healthcare, Pollards Wood, UK).

### Immunofluorescence of adult gonads

For observation of endogenous W10G11.20/DNC-3 in adult gonads using anti-W10G11.20/DNC-3 antibody, young adult worms of the wild type (N2) were paralyzed on MAS-coated glass slides (MATSUNAMI, Osaka, Japan), and the head regions were cut off at the level of the pharynx using a razor. Gonad arms that were extruded from the worm body and attached to the glass slide were used for immunofluorescence observation. Gonads were fixed and stained as described (Sumiyoshi *et al.* 2002) by using a 1 : 200 dilution of mouse anti-KT23 to stain nuclear envelope (Takeda *et al.* 2008) or a 1 : 300 dilution of rabbit anti-W10G11.20/DNC-3. The secondary antibodies used were a 1 : 500 dilution of Alexa- 488-conjugated goat anti-mouse IgG and a 1 : 500 dilution of Alexa-568-conjugated goat anti-rabbit IgG (Invitrogen). Slides were counterstained with 2 mg/mL DAPI. For the image acquisition, the same setting with the live imaging was used.

### Construction of plasmids for expression of GST-fused dynactin

The DNC-1, 2 and 3 coding DNA fragments were amplified from a cDNA library and cloned into pENTR201 (Invitrogen). N- or C-terminal truncated DNC-3 was made as follows. The truncation point was determined by changes of secondary structure predicted by Chou–Fasman analysis (Chou & Fasman 1978). The PCR-amplified DNC-3N (1–71) or DNC-3C (72–171) cDNA fragments were cloned into the pDONR201 entry vector via BP recombination. The entry clones were recombined into pDEST15 for GST or pDEST17 for 6xHis (Invitrogen) via LR reaction.

### GST pull-down assay and immunoblotting

The GST-tagged proteins were expressed in 3 mL of *E. coli* culture and lysed by sonication in 1 mL of binding buffer (50 mm Tris–HCl (pH7.4), 300 mm NaCl, 0.01% Tween20, 1 mm dithiothreitol) containing a protease inhibitor cocktail (Roche) supplemented with 1 mm PMSF. The lysates were centrifuged at 20 000 *g* for 10 min, and the supernatant were incubated for 2 h at 4 °C with MagneGST Glutathione Particles (Promega, Madison, WI, USA). After washing with binding buffer four times, approximately 100 μg of GST-tagged proteins that bound to MagneGST Glutathione Particles were used in each assay. 6xHis-tagged proteins were also expressed in 3 mL of *E. coli* culture, and the lysates were prepared as described for GST-tagged proteins. One hundred microliters of the lysate was added to the GST-tagged proteins that immobilized on MagneGST Glutathione Particles, then incubated for 2 h at 4 °C. After washing with binding buffer four times, GST-tagged proteins and interactors were eluted by 100 mm reduced glutathione. The eluted proteins were separated by SDS–PAGE and detected by immunoblotting. Immunoblotting were carried out by SNAPi.d. (Millipore, Billerica, MA, USA). All primary antibodies, anti-DNC-1 (gift from Dr. Skop) or anti-His-tag (Abgent, San Diego, CA, USA) for detection of DNC-2, were diluted 1 : 1000, the HRP-conjugated goat anti-rabbit secondary antibody (GE Healthcare) was diluted 1 : 5000 and the signal was detected by using ECL plus chemiluminescence (GE Healthcare).

## Supporting Information/Supplementary material

The following Supporting Information can be found in the online version of the article:

**Figure S1** Localization of W10G11.20 in adult hermaphrodite gonads.

**Figure S2** DNC-3/W10G11.20 was co-immunoprecipitated with DNC-1, GFP::DNC-2 and GFP::ARP-1.
